# Multiple biomarker tissue arrays: A computational approach to identifying protein-protein interactions in the EGFR/ERK signalling pathway

**DOI:** 10.1186/1750-2187-7-14

**Published:** 2012-09-01

**Authors:** V Medina Villaamil, G Aparicio Gallego, M Valladares-Ayerbes, I Santamarina Caínzos, L Miguel Antón Aparicio

**Affiliations:** 1 INIBIC, Oncology Group, CHU A Coruña, A Coruña, Coruña, Spain; 2 Medical Oncology Service, CHU A Coruña, A Coruña, Coruña, Spain; 3 UDC Medical Department, A Coruña, Coruña, Spain

**Keywords:** EGFR, Interacting proteins, Renal cell carcinoma, Tissue array

## Abstract

**Background:**

Many studies have demonstrated genetic and environmental factors that lead to renal cell carcinoma (RCC) and that occur during a protracted period of tumourigenesis. It appears suitable to identify and characterise potential molecular markers that appear during tumourigenesis and that might provide rapid and effective possibilities for the early detection of RCC. EGFR activation induces cell cycle progression, inhibition of apoptosis and angiogenesis, promotion of invasion/metastasis, and other tumour promoting activities. Over-expression of EGFR is thought to play an important role in tumour initiation and progression of RCC because up-regulation of EGFR has been associated with high grade cancers and a worse prognosis.

**Methods:**

Characterisation of the protein profile interacting with EGFR was performed using the following: an immunohistochemical (IHC) study of EGFR, a comprehensive computational study of EGFR protein-protein interactions, an analysis correlating the expression levels of EGFR with other significant markers in the tumourigenicity of RCC, and finally, an analysis of the utility of EGFR for prognosis in a cohort of patients with renal cell carcinoma.

**Results:**

The cases that showed a higher level of this protein fell within the clear cell histological subtype (p = 0.001). The EGFR significance statistic was found with respect to a worse prognosis. *In vivo* significant correlations were found with PDGFR-β, Flk-1, Hif1-α, proteins related to differentiation (such as DLL3 and DLL4 ligands), and certain metabolic proteins such as Glut5. *In silico* significant associations gave us a panel of 32 EGFR-interacting proteins (EIP) using the APID and STRING databases.

**Conclusions:**

This work summarises the multifaceted role of EGFR in the pathology of RCC, and it identifies EIPs that could help to provide mechanistic explanations for the different behaviours observed in tumours.

## Background

The ErbB family of receptor tyrosine kinases (RTKs) couples the binding of extracellular growth factor ligands to intracellular signalling pathways regulating diverse biological responses, including proliferation, differentiation, cell motility, and survival. Ligand binding to the four closely related members of this RTK family—epidermal growth factor receptor (EGFR, also known as ErbB-1 or HER1), ErbB-2 (HER2), ErbB-3 (HER3), and ErbB-4 (HER4)—induces the formation of receptor homo- and hetero-dimers and the activation of the intrinsic kinase domain.

The Shc- and/or Grb2-activated mitogen-activated protein kinase (MAPK) pathway is a common target downstream of all of the ErbB receptors. Similarly, the phosphatidylinositol-3-kinase (PI-3 K) pathway is directly or indirectly activated by most of the ErbBs. Several cytoplasmic docking proteins appear to be recruited by specific ErbB receptors and are less exploited by others. These include the adaptors Crk and Nck, the phospholipase C gamma (PLCgamma), the intracellular tyrosine kinase Src, or the Cbl E3 ubiquitin protein ligase [1].

**Figure 1:**
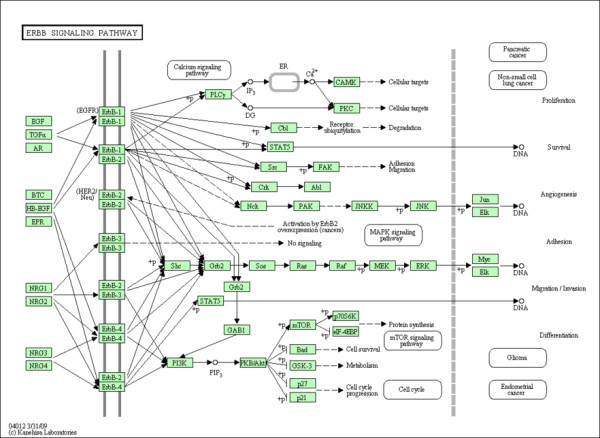
**ERBB Pathway.** The ERBB Signalling Pathway obtained from the Kyoto Encyclopedia of Genes and Genomes (KEGG).

EGFR signalling cascade is one of the best studied and most important signalling pathways in mammals. This pathway regulates cell growth, survival, proliferation and differentiation (Figure 1). EGFR signalling is critically involved in renal organogenesis and electrolyte homeostasis [2]. 

Multiple studies have shown over-expression of the EGFR receptor in renal cell carcinoma (RCC) compared with normal renal tissue, and EGFR expression in RCC was localized to the cell membrane, whereas the EGFR expression in normal kidney tissues was chiefly observed in the cytoplasm, and this different location of EGFR expression could be associated with human renal tumourogenesis [3]. EGFR over-expression is thought to play an important role in tumour initiation and progression of RCC because the up-regulation of EGFR has been associated with a high grade and a worse prognosis [4]. Moreover, clear-cell RCC is frequently associated with the loss of von Hippel-Lindau (VHL) tumour suppressor gene function, which results in the aberrant transcriptional activation of genes that contribute to tumour growth and metastasis. Tumour hypoxia, independent of VHL loss of function, increases EGFR expression through early growth response factor 1 (Egr-1) [5].

Despite some great successes, many human diseases cannot yet be effectively treated, prevented or cured. Hence, there is a need to investigate the molecular basis of these diseases in more detail. For this purpose, relevant biomedical data must be gathered, integrated and analysed in a meaningful way. Mechanistic understanding requires the integration of all of the information that is available about the involved key players and how they interact within the cell. These interactions are typically represented by means of biological networks. The biological context in which disease-related genes operate must be considered. Many human diseases cannot be attributed to the malfunction of single genes but instead arise from complex interactions among multiple genetic variants [6]. Hence, to understand disease mechanisms, a network of key players that are related to the disease and their interactions, for example through biological pathways, must be considered. A biological pathway can circumscribe several types of biological processes, including regulatory, metabolic and signalling processes or protein-protein interactions (PPI). The purpose of this work is to probe in-depth into the EGFR signalling pathway and EGFR PPIs in an RCC population. Although several bioinformatic studies have been undertaken for similar purposes [7-9], we consider that more effort based on validated experimental information is needed to improve the quality of the PPIs that can be obtained from the interactome networks.

## Materials and methods

### Case selection

The patient cohort included 80 patients who were treated with a partial or radical nephrectomy for RCC, including chromophobe RCC (chRCC), papillary RCC (pRCC) and clear-cell RCC (cRCC) variants, and who were recruited between 1996 and 2006. Immunohistochemistry (IHC) studies were performed and clinical data from an established kidney cancer database were reviewed. The Institutional Review Board of Modelo Hospital approved the retrospective review of the medical records and the use of archived tumour specimens.

**Table 1:** List of antibodies

**ANTIBODY & EGFR correlationp-value**	**COMPANY**	**DILUTION**	**PRETREATMENT**	**POSITIVE CONTROL**
**Hif1-α (p = 0.046)**	Abcam	1:1500	Citrate	Squamous cell Carcinoma
**VHL** (p = 0.114)	ABR	Ready to use	Citrate	Healthy Kidney
**VEGF** (p = 0.755)	Sta Cruz	1:100	Citrate	Healthy Kidney
**Flk-1 (p = 0.001)**	Sta Cruz	1:50	Citrate	Healthy Kidney
**CAIX** (p = 0.538)	Abcam	1:500	Citrate	Renal cell carcinoma
**EGFR**	Dako	1:25	Proteinase K	Squamous cell Carcinoma
**PDGFR-α** (p = 0.320)	Sta Cruz	1:200	Citrate	Ovarian Carcinoma
**PDGFR-β (p = 0.000)**	Serotec	1:40	EDTA	Breast Carcinoma
**TGF-α **(p = 0.260)	LabVision	Ready to use	Citrate	Anterior Pituitary
**TGF-β** (p = 0.456)	Millipore	1:500	Citrate	Colon Carcinoma
**C-KIT** (p = 0.182)	Dako	1:50	No required	GIST
**NOTCH1** (p = 0.052)	Abcam	3ug/ml	Citrate	Healthy Kidney
**NOTCH2** (p = 0.937)	Lifespan	1:250	Citrate	Healthy Kidney
**NOTCH3 **(p = 0.249)	Sta Cruz	1:50	Citrate	Healthy Kidney
**NOTCH4 **(p = 0.337)	Sta Cruz	1:100	Citrate	Healthy Kidney
**JAGGED1** (p = 0.058)	Sta Cruz	1:50	EDTA	Astrocytoma
**DLL1** (p = 0.175)	Serotec	10ug/ml	EDTA	Healthy Kidney
**DLL3 (p = 0.049)**	Aviva	15ug/ml	Citrate	Healthy Kidney
**DLL4 (p = 0.046)**	Serotec	10ug/ml	Citrate	Healthy Kidney
**p53** (p = 0.696)	Dako	1:50	EDTA	Breast Carcinoma
**BAX** (p = 0.077)	Abcam	Ready to use	EDTA	Amygdala
**MDM2** (p = 0.394)	Abcam	1.5ug/ml	Citrate	Squamous cell Carcinoma
**Survivin (p = 0.218)**	Sta Cruz	1:100	Citrate	Breast Carcinoma
**BCL2 (p = 0.000)**	Dako	1:75	EDTA	Amygdala
**Glut-1** (p = 0.296)	Abcam	Ready to use	Citrate	Healthy Esophagous
**Glut-2** (p = 0.328)	Sta Cruz	1:50	Citrate	Liver
**Glut-3** (p = 0.529)	Abcam	1:25	Citrate	Placenta
**Glut-4** (p = 0.933)	Abcam	1:250	Citrate	Hearth
**Glut-5 (p = 0.006)**	Abcam	1:250	Citrate	Healthy Small intestine

### Immunohistochemistry tissue array analysis related to renal cell carcinoma

All of the archival tissue samples were routinely stored in formalin and were embedded in paraffin. The representative tissue areas were marked on standard hematoxylin and eosin (H&E) sections, were punched out of the paraffin block using a 2.0-mm punch and were inserted in a recipient paraffin block to produce a 6x8 array of 48 cases. A normal cerebellum tissue sample was inserted as a negative control. When possible, triplicate cores per specimen were arrayed on a recipient paraffin block to decrease the error that was introduced by sampling and to minimise the impact on the tissue during processing. Sections (4 μm) were cut from the completed array blocks and were transferred to silanised glass slides. The primary antibodies used are listed in Table 1. The immunohistochemistry (IHC) technique was conducted, as previously described [10]. The expression was evaluated in a blinded fashion to validate the diagnostic morphology of each array spot. The evaluation of the expression involved the site and the degree of reactivity. The site of the reactivity included the evaluation of the relevant histological subtype as well as the subcellular localisation. The degree of reactivity included the evaluation of the maximal staining intensity using a 0 to 3 scale (0, negative; 1, weak; 2, moderate; 3, strong) as well as the percentage of positive cells at each stated intensity. The pathological variables that were studied are listed in Table 2. 

**Table 2:** Pathological variables analysed in this study

**PATHOLOGICAL**	**PATIENTS**	**RELATED MARKER**
**VARIABLES**	**NUMBER N = 80**	
Differentiation degree (Fuhrman Grade)		
Well	15 (18.75%)	
Moderate	45 (56,25%)	
Poor	16 (20%)	
Undifferentiated	4 (5%)	EGFR p = 0.081
Pelvis invasion		
Yes	9 (11,25%)	EGFR p = 0.014
No	66 (82,5%)	
Undetermined	5 6,25%)	
Breaking capsule		
Yes	12 (15%)	EGFR p = 0.008
No	64 (80%)	
Undetermined	4 (5%)	
Tumor depth		
1	63 (78,75%)	
2	4 (5%)	
3	12 (15%)	EGFR p = 0.833
4	1 (1,25%)	
Histology type		
Clear cell	57 (71,25%)	EGFR p = 0.001
Papillary	6 (7,5%)	
Chromophobe	15 (18,75%)	
Undetermined	2 (2,5%)	
Tumor localization		
Right	40 (50%)	EGFR p = 0.275
Left	39 (48,75%)	
Undetermined	1 (1,25%)	
Veins invasion		
Yes	9 (11,25%)	EGFR p = 0.475
No	67 (83,75%)	
Undetermined	4 (5%)	
Lymphatic vessels invasion		
Yes	2 (2,5%)	EGFR p = 0.040
No	78 (97,5%)	
Hilar invasion		
Yes	6 (7,5%)	EGFR p = 0.029
No	68 (85%)	
Undetermined	6 (7,5%)	
Node involvement		
0	19 (23,75%)	
1–5	59 (73,75%)	
>5	2 (2,5%)	EGFR p = 0.000

### PPI resources

One of the most productive areas of current research is the area of protein-protein interactions and interactome data [11]. Data about the interaction of two or more proteins come either from small-scale experimental work or from large-scale experimental methods. Protein interaction resources include the following 2 databases:

1. Agile Protein Interaction DataAnalyser (APID) [12]: developed to better assess the quality of the PPI data and to provide a more comprehensive integration of the main currently known PPI interactions. APID integrates data coming from five main source databases: BIND [13], DIP [14], HPRD (Human protein reference database) [15], IntAct (Database system and analysis tools for protein interaction data) [16] and MINT (Molecular Interactions Database) [17].

2. Search Tool for the Retrieval of Interacting Genes (STRING) [18]: an update to provide all of the information on functional links between proteins. The main strengths of STRING lie in its unique comprehensiveness, its confidence scoring and its interactive and intuitive user interface. All of the associations in STRING are provided with a probabilistic confidence score, which is derived by separate groups of associations from the manually curated functional classification scheme of the KEGG database [19].

### Human EGFR protein network prediction

Predictions of EPI have been attached to obtain insights about mechanisms of disease development and to find key proteins that are related to a disease or a biological pathway [20]. There are computational tools to predict PPIs, such as gene neighbourhood [21], gene fusion [22], phylogenetic profile [23], and interolog [24]. In the interolog approach, the interaction of 2 query proteins is predicted when both have homologous proteins that are already known to interact [20]. We performed a STRING search in the protein mode. The prediction methods that were activated are the following: neighbourhood, gene fusion, co-occurrence, co-expression, experiments, databases, text mining and homology. The number of associations stored in STRING was shown separately for each data source and confidence range (low: scores < 0.4, medium: scores from 0.4 to 0.7, and high: scores > 0.7). Only those with a high confidence were accepted.

### Statistical analysis methods

Data are expressed as the mean ± the standard deviation (SD). The non-normality of the distribution of the protein expression values was assessed by the Kolmogorov-Smirnov test. Thus, non-parametric statistics (Mann–Whitney and Kruskal-Wallis test) were used to analyse the potential correlation between protein expression and the pathological features of the study subjects. P values < 0.05 were considered to be significant. The standard Pearson correlation values for IHC data of the molecular factors studied were calculated. All of the statistical analyses were performed using commercially available software (SPSS 19.0 for Windows).

## Results

### Immunohistochemical staining of EGFR and its correlation with pathological variables

The cases that showed higher membranous positivity for this protein were those falling within the clear cell histological subtype (p = 0.001). Those cases expressing more EGFR were consistent with pathological features associated with a worse prognosis: pelvic (p = 0.014), and lymphatic vessels invasion (p = 0.040), rupture of the renal capsule (p = 0.008), renal hilar invasion (p = 0.029) and greater node involvement (p = 0.001). We found no statistical support to associate this protein with other pathological variables that were analysed.

### The correlation of EGFR protein with other markers

**Figure 2:**
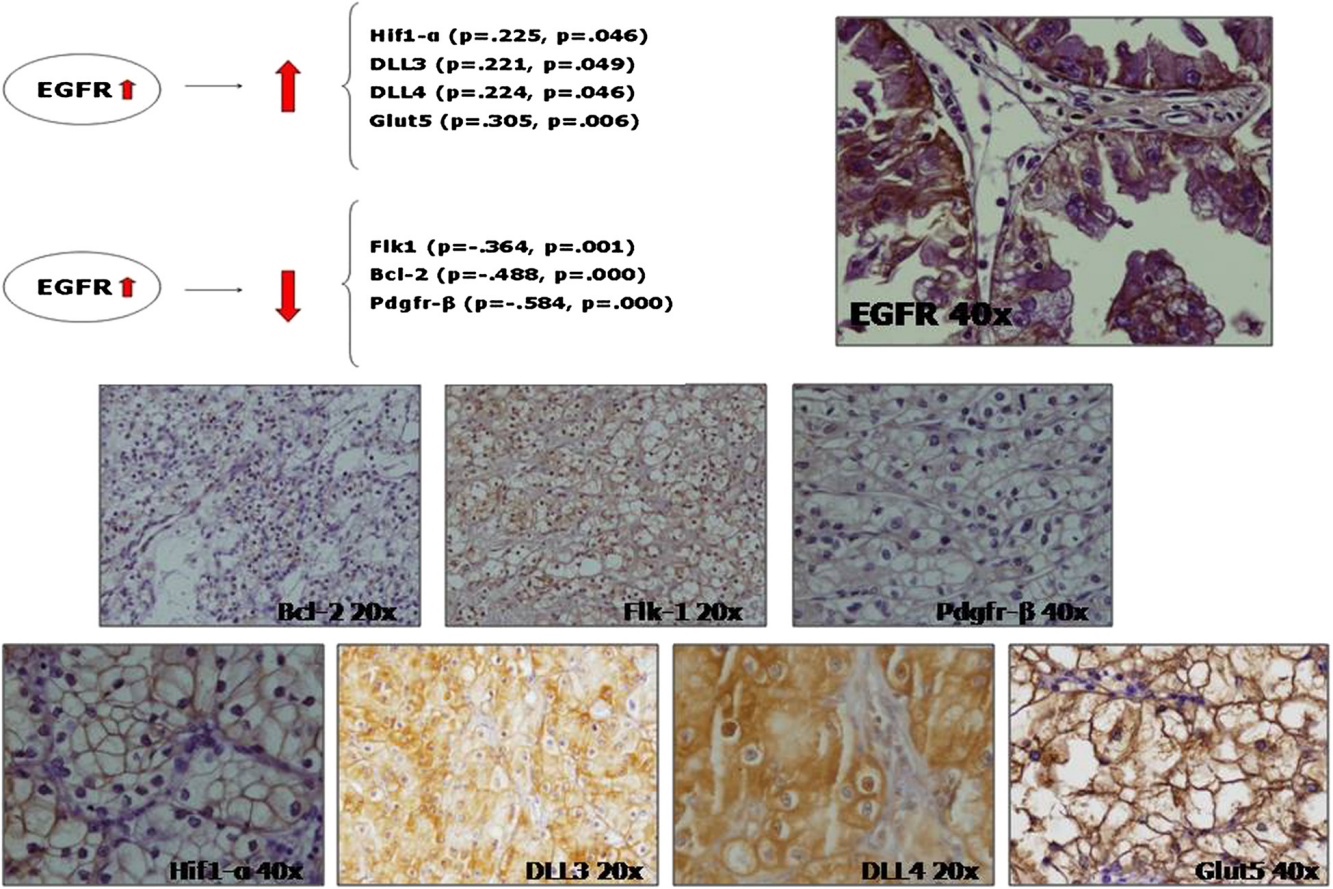
**Immunohistochemistry of those EGFRs that are statistically associated proteins.** Proteins with statistically significant associations are represented with their statistical values (Pearson correlation and p-value associated) and by immunohistochemistry staining.

The statistical significance values of correlations between molecular variables are listed in Figure 2. The analysis of the relationship between EGFR and a panel of relevant RCC tumour markers (see Table 1) revealed a significant correlation with receptor tyrosine kinases, such as vascular endothelial growth factor receptor 2 (Flk1) and platelet derived growth factor receptor beta (PDGFR-β), hypoxia-inducible factor 1-alpha (Hif1-α), apoptosis regulator Bcl-2 (Bcl-2), proteins related to differentiation, such as delta-like protein 3 and 4 (DLL3 and DLL4), and proteins related to fructose uptake such as facilitated fructose transporter, member 5 (Glut5). Our results indicate that EGFR is statistically significantly associated with 7 of the 29 molecules studied, an increase or decrease of EGFR expression may account for the level of expression of each of the following 7 proteins. The Pearson statistic indicated that the strongest positive association is found with the fructose transporter Glut5 followed by Hif1-α and the ligands DLL4 and DLL3, all of which had Pearson values that were very similar and indicative of a weaker association. On the other hand, the Pearson statistic showed a strong negative trend of association between EGFR and the growth factor PDGFR-β followed by the anti-apoptotic Bcl-2, finally EGFR displayed a moderate negative association with Flk1. Figure 2 shows the IHQ expression of EGFR and the associated proteins with Pearson statistic values.

### PPI by APID

**Figure 3:**
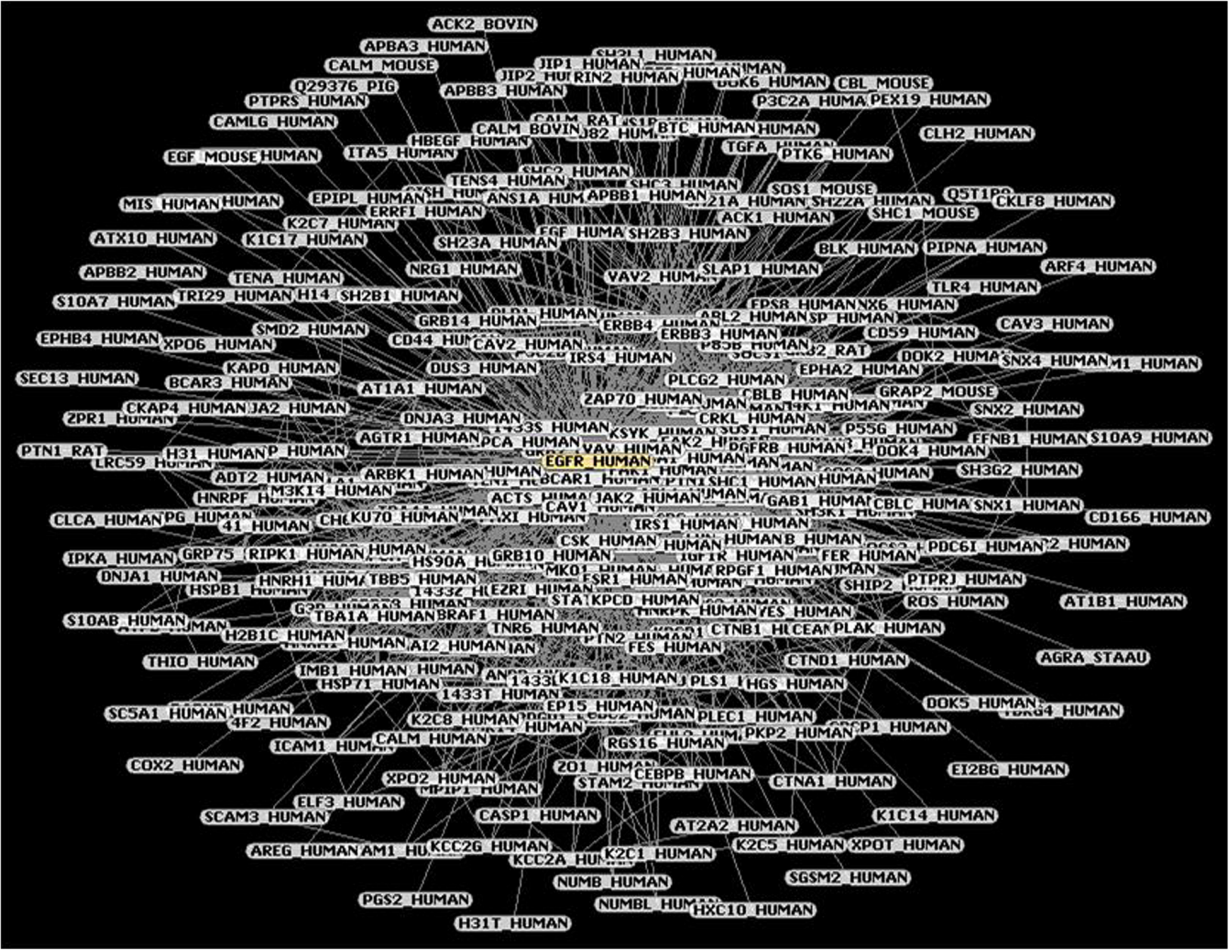
**APID Network.** Proteins displayed by APID without filters. Proteins are more or less close to EGFR according to their cluster coefficient.

The starting query EGFR_HUMAN gave us a sample table with only one row because only one protein was found. The program displayed 282 proteins that interact with EGFR (Figure 3). The selectors presented allow us to filter the data to choose only the interactions that are validated by at least a certain number of experiments that prove a protein-protein interaction, or to filter the data to choose only the interactions that are supported by the presence in the protein pair of two Pfam domains that are known to interact according to the 3D structural interaction database iPfam. With the intention of tightening the search, we stayed with only those interactions that were verified by more than three experiments and that had iPfam validation. In this way, the number of interaction partners for EGFR was reduced to only 21 proteins, which are detailed in Table 3. Thus, we can say that the growth factor receptor-bound protein 2 (GRB2), epidermal growth factor (EGF), (Src homology 2 domain containing) transforming protein 1 (SHC1), Ras GTPase-activating protein 1 (RASA1), Proto-oncogene C-crk (CRK) and 1-phosphatidylinositol-4,5-bisphosphate phosphodiesterase gamma-1 (PLCG1) proteins are the most experimentally studied proteins in relation to EGFR interactions, which has allowed us to improve our knowledge of the EGFR interactome network.

**Table 3:** EGFR interaction partners obtained by APID

**PROTEIN INTERACTORS**	**EXPERIMENTS**	**PROVENANCE**	**CLUSTER COEFFICIENT**
EGFR_HUMAN/GRB2_HUMAN	18	BIND - HPRD - IntAct - MINT - BioGRID	0.018153
EGF_HUMAN/EGFR_HUMAN	15	BIND - DIP - HPRD - IntAct - MINT - BioGRID	0.242424
EGFR_HUMAN/SHC1_HUMAN	12	BIND - DIP - HPRD - IntAct - MINT - BioGRID	0.100999
PTN1_HUMAN/EGFR_HUMAN	9	HPRD - MINT - BioGRID	0.070715
EGFR_HUMAN/RASA1_HUMAN	7	BIND - HPRD - MINT - BioGRID	0.122177
CRK_HUMAN/EGFR_HUMAN	6	HPRD - MINT - BioGRID	0.049001
SRC_HUMAN/EGFR_HUMAN	5	HPRD - MINT - BioGRID	0.033376
PLCG1_HUMAN/EGFR_HUMAN	5	BIND - HPRD - MINT - BioGRID	0.064601
EGFR_HUMAN/STAT3_HUMAN	4	BIND - HPRD - IntAct - BioGRID	0.078719
EGFR_HUMAN/JAK2_HUMAN	4	HPRD - MINT - BioGRID	0.117345
NCK1_HUMAN/EGFR_HUMAN	4	HPRD - MINT - BioGRID	0.022317
GRB10_HUMAN/EGFR_HUMAN	4	HPRD - MINT - BioGRID	0.22792
TGFA_HUMAN/EGFR_HUMAN	3	BIND - DIP - HPRD - IntAct - BioGRID	0.044118
PTN11_HUMAN/EGFR_HUMAN	3	HPRD - MINT - BioGRID	0.09284
SH3K1_HUMAN/EGFR_HUMAN	3	HPRD - MINT - BioGRID	0.108213
P85A_HUMAN/EGFR_HUMAN	3	BIND - HPRD - MINT	0.047572
EGFR_HUMAN/STAT1_HUMAN	3	HPRD - BioGRID	0.080438
EGFR_HUMAN/VAV2_HUMAN	3	HPRD - BioGRID	0.235294
EGFR_HUMAN/STA5A_HUMAN	3	HPRD - BioGRID	0.193759
SOS1_HUMAN/EGFR_HUMAN	3	HPRD - BioGRID	0.155465
EGFR_HUMAN/PTN6_HUMAN	3	HPRD - BioGRID	0.101232

The table shows 21 proteins with greater evidence for EIP (EGFR interacting proteins). The cluster coefficient, a graph parameter that indicates the degree of inter-connection of the group of proteins directly interact to a query protein, in our case, EGFR. A cluster coefficient value close to zero indicates that the protein pair is very close, a value away from zero shows that the protein pair is farthest.

### PPI by STRING

**Figure 4:**
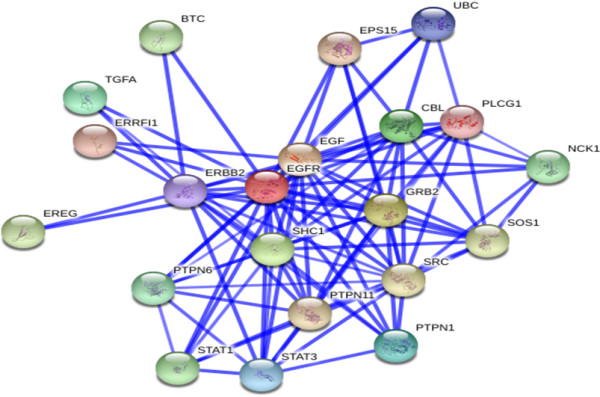
**STRING Network.** EGFR interacting protein network obtained by STRING. This view is the confidence view. Stronger associations are represented by thicker lines.

The EGFR protein network obtained from the STRING database was formed for the next proteins: EGF, GRB2, SHC1, cas-Br-M (murine) ecotropic retroviral transforming sequence (CBL), transforming growth factor alpha (TGFA), protein tyrosine phosphatase, non-receptor type 1 (PTPN1), signal transducer and activator of transcription 3 (acute-phase response factor) (STAT3), ubiquitin C (UBC), v-erb-b2 erythroblastic leukemia viral oncogene homolog 2 (ERBB2), phospholipase C, gamma 1 (PLCG1), ERBB receptor feedback inhibitor 1 (ERRFI1), epidermal growth factor receptor pathway substrate 15 (EPS15), protein tyrosine phospatase, non-receptor type 11 (PTPN11), v-src sarcoma (Schmidt-Ruppin A-2) viral oncogene homolog (avian) (SRC), son of sevenless homolog 1 (Drosophila) (SOS1), epiregulin (EREG), betacellulin (BTC), signal transducer and activator of transcription 1 (STAT1), NCK adaptor protein 1 (NCK1) and protein tyrosine phosphates, non-receptor type 6 (PTPN6). All of these proteins had the required confidence (score) higher than 0.7, and no more than 20 interactors were shown. Figure 4 shows the STRING EIP network. Table 4 shows the data scores for the interactors.

**Table 4:** EGFR scores for the interactors obtained by STRING

**Node 1 Node 2 Combined score**			**Node 1 Node 2 Combined score**		
UBC	PLCG1	0.845	SOS1	PTPN11	0.999
*STAT1	**EGFR**	**0.998**	SHC1	PTPN1	0.920
SOS1	GRB2	0.999	**PTPN6**	**EGFR**	**0.998**
GRB2	PLCG1	0.992	**EGFR**	*PLCG1	**0.999**
EPS15	EGF	0.983	SRC	ERBB2	0.855
EGF	STAT3	0.996	PTPN1	STAT3	0.966
ERRFI1	EGF	0.956	SRC	EGF	0.999
**PTPN1**	**EGFR**	**0.999**	STAT1	STAT3	0.996
BTC	ERBB2	0.990	ERBB2	EGF	0.999
UBC	EGF	0.922	GRB2	CBL	0.999
SRC	PLCG1	0.989	SHC1	STAT3	0.869
SRC	NCK1	0.931	NCK1	CBL	0.948
ERBB2	EREG	0.966	SOS1	CBL	0.972
GRB2	ERBB2	0.999	EPS15	GRB2	0.992
**EPS15**	**EGFR**	**0.999**	**EGFR**	*STAT3	**0.999**
SHC1	TGFA	0.724	SRC	GRB2	0.992
EGF	CBL	0.980	PTPN6	STAT1	0.781
SOS1	SRC	0.986	**EGFR**	**EREG**	**0.999**
GRB2	STAT3	0.973	SHC1	SRC	0.806
GRB2	EGF	0.998	*GRB2	**EGFR**	**0.999**
PTPN6	CBL	0.714	ERRFI1	ERBB2	0.933
SHC1	PLCG1	0.913	EPS15	UBC	0.995
PTPN1	SRC	0.999	CBL	PLCG1	0.999
SHC1	ERBB2	0.998	PTPN1	ERBB2	0.884
PTPN11	ERBB2	0.990	SHC1	PTPN6	0.766
**EGFR**	*EGF	**0.999**	SHC1	CBL	0.999
TGFA	ERBB2	0.992	PTPN11	CBL	0.960
**EGFR**	**CBL**	**0.999**	PTPN6	GRB2	0.969
NCK1	PLCG1	0.708	**ERRFI1**	**EGFR**	**0.999**
PTPN1	GRB2	0.999	PTPN6	EGF	0.976
PTPN11	GRB2	0.999	*SRC	**EGFR**	**0.999**
SHC1	EGF	0.996	SOS1	ERBB2	0.961
SRC	STAT3	0.999	STAT1	PTPN11	0.998
NCK1	EGF	0.979	SHC1	PTPN11	0.971
**EGFR**	**ERBB2**	**0.999**	STAT1	SRC	0.992
SRC	PTPN11	0.802	PTPN11	EGF	0.996
*SOS1	**EGFR**	**0.999**	EPS15	SRC	0.807
SOS1	PLCG1	0.943	SHC1	STAT1	0.830
PTPN6	STAT3	0.893			
PTPN11	PLCG1	0.727			
PTPN11	STAT3	0.988			
**UBC**	**EGFR**	**0.999**			
UBC	CBL	0.995			
ERBB2	PLCG1	0.982			
EGF	PLCG1	0.981			
EPS15	CBL	0.960			
ERBB2	STAT3	0.989			
PTPN1	EGF	0.979			
PTPN6	PTPN11	0.961			
**SHC1**	**EGFR**	**0.999**			
PTPN6	SRC	0.780			
**BTC**	**EGFR**	**0.999**			
*PTPN11	**EGFR**	**0.999**			
SOS1	NCK1	0.998			
STAT1	EGF	0.995			
*NCK1	**EGFR**	**0.998**			
*TGFA	**EGFR**	**0.999**			
SOS1	EGF	0.971			
SRC	CBL	0.998			
SHC1	SOS1	0.999			
SHC1	GRB2	0.999			

The table shows all associations in STRING provided with a probabilistic confidence score for EIP and between proteins belongs to this EGFR interactome network. Each node represents a protein which by edges may be interacting with any other. Each score represents a rough estimate of how likely a given association describes a functional linkage between two proteins that is at least as specific as that between an average pair of proteins annotated on the same ‘map’ or ‘pathway’ in KEGG. *Proteins showed by STRING and APID as EIP.

## Discussion

The majority of human epithelial cancers are marked by the activation of EGFR, which was the first growth factor receptor to be proposed as a target for cancer therapy. Dysregulation of EGFR is often observed in association with carcinogenesis, which can be caused by receptor over-expression, mutations or deletions [25]. A blockade of EGFR results in the inhibition of growth in several human carcinoma cell lines [26]. Over-expression of EGFR and its family members have been found in the majority of human cancers. Cancer patients with EGFR over-expression often have a worse prognosis [27]. The majority of human carcinomas can synthesise and secrete EGF-like growth factors that can bind to ErbB receptors expressed in accessory cells of the tumour microenvironment [28]. This interaction has been shown to regulate important mechanisms of tumour progression, such as the proliferation and motility of endothelial cells and the production of pro-angiogenic and proosteoclastogenic cytokines in tumour and stromal cells. This observation of EGFR makes an interesting target for therapeutic intervention even in tumours with EGFR-independent growth. Interestingly, the clinical activity of anti-EGFR agents in patients carrying EGFR-negative tumours has already been demonstrated [29].

Our tissue array study demonstrated that the increased expression of EGFR was a dependent predictor of a worse prognosis for patients with RCC. Moreover, increased EGFR staining was associated with the clear cell histological subtype. Our results were identical with previous studies that showed that a higher expression of membranous EGFR was frequently detected [27].

Previous studies have shown EGFR over-expression in the advanced stage, poor prognosis and metastatic human cancer [30]. Over-expression of EGFR played an important role in tumour initiation and progression of RCC; thus, the up-regulation of EGFR was correlated with high-grade tumours and a worse prognosis [31].

The prognostic association of EGFR over-expression in RCC, however, is a controversial issue. Some studies showed an association of EGFR immunoreactivity with well differentiated RCCs [32] or regarded strong membranous EGFR immunostaining as an indicator of good prognosis [33], whereas others showed an association of EGFR immunoreactivity with high tumour stage/grade and poor prognosis [34] or showed no significant associations at all [35]. As expected from previous studies [27], our study showed that there was a significant correlation between the level of membranous EGFR expression and the histologic subtype, with a higher expression in conventional RCC compared to non-conventional RCC (including papillary and chromophobe).

Biostatistical analysis of the IHC scores obtained from 29 markers studied in samples of patients affected by renal tumours gave us an EGFR positive association, an increase of EGFR means an increase in the next proteins and vice versa, with Hif1-α, DLL3, DLL4 and Glut5 and an EGFR negative association, an increase of EGFR means a decrease in the next proteins and vice versa, with Flk1, Bcl-2 and PDGFR-β.

Previous studies in RCC cells link HIF activation with the aberrant production of a bona fide mitogen of renal epithelial cells and provide evidence for a role of HIF in the initiation of tumourigenesis [36].

The Notch pathway plays a central role in stem cell maintenance, cell fate decisions, and cell survival. Four members of the Notch family have been identified, each a single-pass transmembrane protein with complex extracellular and intracellular domains. The binding of a Delta-like (DLL1, DLL3 and DLL4 studied in this work) or Jagged (Jagged1) ligand on one cell to Notch on an adjacent cell triggers enzymatic cleavages, which liberate the Notch intracellular domain. We demonstrated previously the presence of Notch1-4 and its ligands DLL1, DLL3, DLL4 and Jagged1 in RCC and their importance [37]. No previous report to our knowledge has demonstrated a major increase in DLL3 and DLL4 expression with increased EGFR. Precedent reports [38] indicate that Notch signalling can have very different outputs depending on whether the ligands are binding, and this difference could be one explanation for context dependence. It has previously been found that Notch activation is key to maintaining Ras pathway activity, one of the downstream mediators of EGFR [39]. The Notch pathway is ubiquitous in development and cell fate determination, and EGFR plays roles in development as well. This report suggests that EGFR is an intermediate of some of the many roles of Notch in development. It also bases another point of cross talk between the Notch and EGFR–Ras pathways.

Previous studies [40] from our group showed that Glut5 expression associates more strongly with the clear cell RCC subtype. The clear cell subtype of RCC is characterised histologically by a distinctive pale, glassy cytoplasm, and this appearance of clear cell RCC is from abnormalities in the carbohydrate and lipid metabolism; these abnormalities result in glycogen and sterol storage. These data suggest a role for the Glut5 isoform in fructose uptake that takes place in clear cell RCC cells and that subsequently leads to the malignant RCC progression. Here, we analysed for the first time a possible link between fructose metabolism and cell proliferation, which is understood to be an over-expression of EGFR in RCC; we found that both proteins characterise the same histological subtype (clear cell RCC), which shows an increase in the metabolic rate by fructose intake in cells that proliferate more.

Interestingly, further analysis revealed an inverse correlation between EGFR and the vascular endothelial growth factor receptor 2, Flk1. The amounts of Flk1, as determined by IHC, were greatly reduced in those RCC samples with higher EGFR staining. Dysregulation of angiogenesis is implicated in the development of many human cancers, especially in clear cell RCC, a highly vascularised tumour. Our findings could show a negative feedback loop at certain times in those tumoural renal cells with EGFR excess, which leads to a decrease in angiogenesis through a decreased expression of Flk1.

The signal transduction pathways activated by the PDGFR-β are well characterised and resemble those of other receptor tyrosine kinases such as EGFR and the vascular endothelial growth factor receptor. Following its activation, the PDGFR-β stimulates intracellular signalling proteins that include Ras-MAPK, phosphatidylinositol 3-kinase, phospholipase Cγ, and ERK1/2 [41]. An explanation for this fact could be that those cells with activated EGFR pathway proliferation could have an attenuated proliferation pathway through PDGFR-β, resulting in decreased angiogenesis and autocrine growth stimulation. Apoptosis is a genetically controlled mechanism of cell death that is involved in the regulation of tissue homeostasis. Bcl-2 antagonises p53-induced apoptosis and can contribute to chemoresistance [42]. The percentage of cells stained was the greatest in the cases that did not have EGFR staining.

The main limitation of our work is that the approach to the expression of selected markers by means of a tissue array study and IHC has not been combined with molecular biology techniques, such as immunoprecipitation or western blotting. We used IHC of primary tumours from patients to demonstrate for the first time the relevant interactions that are involved in different pathways that regulate RCC cell fates.

The models of EIP by STRING suggest valuable interrelations. In the analysis of putative interactors of EGFR based on the score value, the lowest score value of 0.708 was observed for nodes NCK1 and PLCG1, and different nodes were shown, with the highest score being 0.999. The proteins with high score values exhibit a higher affinity for EGFR than low-score proteins (Table 4).

The results are convincing because the data are taken from different species and are based on a variety of experimental methods, such as yeast-two-hybrid, X-ray crystallography, mass spectroscopy, and affinity purification. The list of proteins obtained by STRING could form a variety of functional connections with each other, including stable complexes, metabolic pathways and a bewildering array of direct and indirect regulatory interactions in our cohort of renal tumours. These connections can be conceptualised as networks, and the size and complex organisation of these networks present a unique opportunity to view a given genome as something more than just a static collection of distinct genetic functions. The ‘network view’ of a genome is increasingly used in many areas of applied biology: protein networks are used to increase the statistical power of human genetics, to aid in drug discovery, to close gaps in metabolic enzyme knowledge and to predict phenotypes and gene functions, to name a few examples [43].

STRING is by no means the only such site: APID enabled us to reduce to 21 the number of EIPs demonstrated by specific small-scale or large-scale experimental methods.

An important next step would be to discover new EIPs that could be extracted from *in vitro* biological networks, such as those that validate, with experimental data from the laboratory, the behaviour of the computational network model obtained for EGFR in this work. From this perspective, this study produces an illustration of biological knowledge of molecular interactions from experimental data.

## Conclusions

This study has shown how computational models can be useful tools for investigating and comparing the biological behaviour of signal transduction pathways because they can advance new hypotheses for explaining the observed biological data and help us to understand the dynamics with respect to how the pathways function.

Proteins such as GRB2, EGF, SHC1, PTN1, RASA1, CRK, SRC, PLCG1, STAT3, JAK2, NCK1, GRB10, TGFA, PTN11, SH3K1, P85A, STAT1, VAV2, STAT5A, SOS1, PTN6, CBL, PTPN1, UBC, ERBB2, PLCG1, ERRFI1, EPS15, PTPN11, EREG, BTC, and PTPN6, which are co-expressed with EGFR, are required to maintain the functional status of the cell environment. The correlation of EGFR with proteins such as HIF1-α, DLL1, DLL3, FLK1, Glut5, Bcl-2 and PDGFR-β, which are involved in cell survival and cell proliferation in RCC, should not be ignored.

## Competing interests

The authors declare that they have no potential conflicts of interest.

## Authors’ contributions

Conception and design: VMV and LMAA. Provision of study materials and patients: LMAA and MV-A. Collection and assembly of data: VMV, GAG, ISC. Data analysis and interpretation: VMV, LMAA, MV-A and GAG. Manuscript writing: VMV and LMAA. All of the authors have read and approved the final manuscript.
